# Neonatal Hemochromatosis: Systematic Review of Prenatal Ultrasound Findings—Is There a Place for MRI in the Diagnostic Process?

**DOI:** 10.3390/jcm12072679

**Published:** 2023-04-03

**Authors:** Adelina Staicu, Roxana Popa-Stanila, Camelia Albu, Alexandra Chira, Roxana Constantin, Dan Boitor-Borza, Mihai Surcel, Ioana Cristina Rotar, Gheorghe Cruciat, Daniel Muresan

**Affiliations:** 1 1st Department of Obstetrics and Gynecology, “Iuliu Haţieganu” University of Medicine and Pharmacy, 400012 Cluj-Napoca, Romania; 2 1st Clinics of Obstetrics and Gynecology Cluj-Napoca, Emergency County Clinical Hospital, 400006 Cluj-Napoca, Romania; 3 Department of Radiology, “Iuliu Haţieganu” University of Medicine and Pharmacy, 400012 Cluj-Napoca, Romania; 4 Centre of Advanced Research Studies, Emergency County Hospital, IMOGEN, 400006 Cluj-Napoca, Romania; 5 Department of Pathology, “Iuliu Haţieganu” University of Medicine and Pharmacy, 400012 Cluj-Napoca, Romania; 6 Department of Internal Medicine, “Iuliu Haţieganu” University of Medicine and Pharmacy, 400012 Cluj-Napoca, Romania; 7 2nd Medical Clinic, Emergency County Clinical Hospital, 400006 Cluj-Napoca, Romania

**Keywords:** hemochromatosis, gestational alloimmune liver disease, liver failure, magnetic resonance imaging, siderosis, iron, prenatal

## Abstract

Neonatal hemochromatosis (NH) is an uncommon, severe disorder that results in fetal loss or neonatal death due to liver failure. NH is currently regarded as the phenotypic expression of gestational alloimmune liver disease (GALD). The diagnosis of NH-GALD is rarely prenatally established. In addition to providing a systematic review of the prenatal features that are identifiable using ultrasound (US) and MRI, we suggest a prenatal diagnosis algorithm for use in suspected NH during the first affected pregnancy. From a total of 586 database entries identified in PubMed, Google Scholar, and ResearchGate, we selected 18 studies published from 1993 to 2021 that reported maternal medical and obstetric history, prenatal ultrasound findings, and postpartum outcomes. We investigated the ultrasound and MRI features of these studies, along with the outcome due to this condition. A total of 74 cases were identified. The main reported prenatal US finding was fetal growth restriction (FGR) (33%), followed by oligohydramnios (13%) and hydrops fetalis (13%), with 13% cases described as uneventful. Other rare prenatal findings were fetal anemia, ascites, and abnormal fetal liver and spleen. Most pregnancies ended with fetal/perinatal death or therapeutic interruption of pregnancy. Favorable evolution with treatment (ensanguine transfusion and intravenous immunoglobulin (IVIG)) was reported for only 7% of fetuses. Using T2-weighted MRI, fetal extrahepatic siderosis confirmed prenatally in two cases and postnatally in 11 cases. IVIG treatment throughout subsequent pregnancies was found to significantly improve fetal prognosis. MRI should be indicated in selected cases of oligohydramnios, fetal hydrops, fetal hepatomegaly, ascites, or unexplained FGR or anemia after ruling out all other more frequently encountered conditions. MRI can be used to detect iron overload in the liver and extrahepatic siderosis.

## 1. Introduction

Neonatal hemochromatosis (NH) (OMIM 231100) is defined as a severe liver illness accompanied by extrahepatic siderosis in newborns [1]. Evidence points to gestational disease, mainly manifested in utero by fetal liver injury, receiving the name of congenital hemochromatosis [2]. While causality was originally thought to lie in iron metabolism defects, it is now known to be an effect of severe fetal injury, with more than 95% of cases being linked to gestational alloimmune liver disease (GALD) [3]. In rare cases, NH may be caused by other conditions, such as certain perinatal infections (parvovirus B19 or cytomegalovirus), GRACILE (growth retardation, amino-aciduria, cholestasis, iron overload, lactic acidosis, and early death) syndrome, mitochondrial deoxyribonucleic acid depletion from deoxyguanosine kinase deficiency gene mutations, trichohepatoenteric syndrome, deficiency of delta 4-3-oxosteroid 5 beta-reductase due to defects in the synthesis of this bile acid, or 21 trisomy [1,4,5].

While the actual incidence of NH is uncertain (a 2018 study estimated the incidence of GALD in the United States to be 4 per 10,000 live births), relevant findings have resulted in reclassification of NH as both congenital and familial [1,6]. Even if preceding pregnancies have resulted in healthy live births, once the alloimmune reaction occurs, up to 90% of the affected women’s further pregnancies will be impacted by fetal liver disease, regardless of changes in the father [3]. Moreover, the affliction does not occur in pregnancies of other maternal siblings [7].

Information regarding fetal hemochromatosis remains scarce. Since its initial description, evidence has largely been gathered from case reports or case series [8].

NH is often only diagnosed at autopsy, and failure to acquire a correct diagnosis in stillbirths or neonatal deaths with liver failure leads to dire consequences for the mother’s future pregnancies [7]. Left untreated, GALD leads to fetal/neonatal death, making it necessary to consider GALD as a possible cause in all cases of liver disease and unexplained stillbirth, neonatal demise, or early infant death [1]. Because of these findings, the course of diagnosis and treatment of NH has shifted from neonatal to in utero [7].

From 18 weeks of gestation, intrauterine signs that could suggest NH are fetal growth restriction (FGR), oligohydramnios, fetal hydrops, fetal hepatomegaly, ascites, prematurity, unexplained anemia, or fetal demise in the late-second and third trimesters [1,3,9]. These signs are observed early in known affected mothers and lead to a clear diagnosis. However, in the absence of positive maternal history, the diagnosis of NH is easily overlooked, as these ultrasound (US) findings are often encountered in more common pathologies. One method that could lead to prenatal diagnosis of NH in the first affected pregnancy is fetal MRI. MRI is an acceptable, non-invasive method that does not involve significant risks and can be used to confirm hepatic and extrahepatic siderosis in utero or in the first postnatal hours [4].

The current review aims to gather the available evidence regarding the antenatal features related to NH-GALD and create an algorithm for potential assessment when there is prenatal suspicion of this rare pathology characterized by a severe prognosis, wherein selected fetuses will be referred for MRI and the initiation of specific treatment where appropriate.

## 2. Materials and Methods

We conducted a literature review on the subject, focusing on reports of patients diagnosed with NH and with confirmed extrahepatic siderosis that reported maternal medical and obstetric history, prenatal ultrasound findings, and postpartum outcome.

Despite the strong impact of this condition on present and future pregnancies, the prenatal findings that might suggest fetal NH-GALD are often non-specific, leading to an infrequent prenatal diagnosis; therefore, we used strict inclusion criteria.

The protocol for systematic review was written in accordance with the Preferred Reporting Items for Systematic Reviews and Meta-Analyses (PRISMA) guidelines [10]. The review was not registered. The literature review was conducted through systematic searches of the PubMed, Google Scholar, and ResearchGate medical databases, independently conducted by A.S. and R.PS, for studies reported from January 1993 to December 2021, with no restrictions on study design. The keyword term used in searching was “neonatal hemochromatosis”. Additional articles were identified from the reference lists cited in the included articles. We used bibliography software (Zotero version 6.0.23) to collect all query entries and to remove duplicates based on the title, author(s), and journal names.

The studies were screened for relevance (abstracts and then full-text articles) based on the PICOS criteria and comprised case reports, case series, and observational studies (study design) of patients diagnosed with NH. These studies reported maternal medical and obstetric history and prenatal ultrasound findings (population), confirmed extrahepatic siderosis (intervention), and the postpartum outcome (outcome).

Papers with no description of prenatal evolution of the pregnancy or cases where extrahepatic siderosis was not confirmed (the absence of NH diagnosis is debatable) were excluded. We considered papers in English, French, and Spanish.

**Figure 1:**
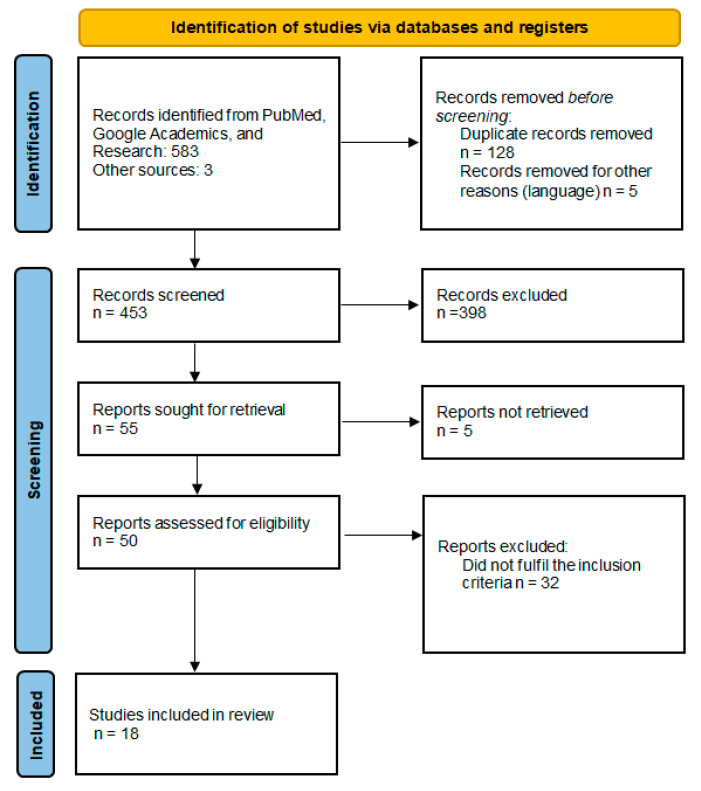
Details of the literature review—PRISMA diagram.

Using the search method, 586 articles were identified from the databases. A total of 128 duplicate articles were removed from the screening, and five were written in languages other than those being considered. Of the 453 articles remaining after screening by title and abstract, 398 were excluded as they referred to another subject. Of the 50 articles assessed for eligibility, 18 met the inclusion criteria (Figure 1).

## 3. Results

The 74 cases of postnatally diagnosed NH with confirmed extrahepatic siderosis found in the current literature review are listed in Table 1 (Table 1). For each case, all the available data are presented, including maternal medical and obstetric history, prenatal findings, postpartum outcome, and subsequent pregnancy outcome, if available. We identified fourteen case reports and three retrospective observational studies.

**Figure 2:**
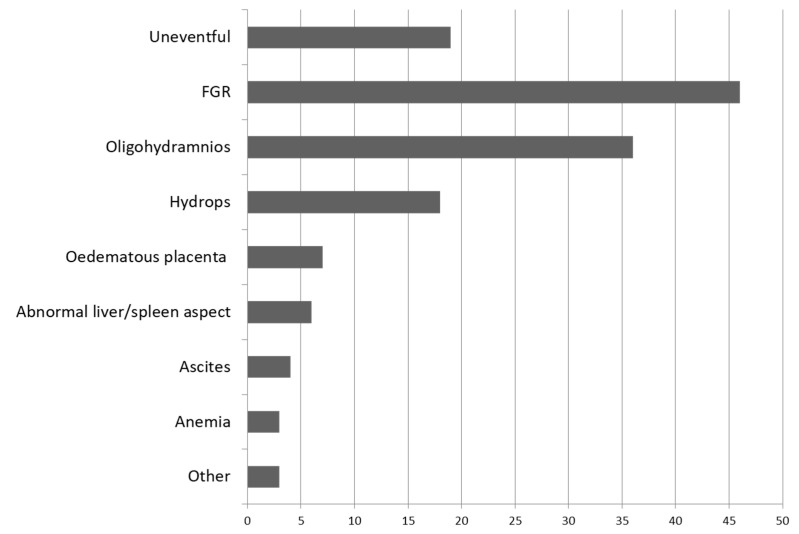
Prenatal findings in 74 cases evaluated in the literature review diagnosed with neonatal hemochromatosis and with confirmed extrahepatic siderosis, reported from 1993 to 2021.

NH was reported to affect mainly multiparous, healthy gravidas at a mean of 33 weeks of gestational age (WGA) (range 21 to 40). A small percentage of the gravidas presented autoimmune pathologies, such as systemic erythematosus lupus (4%), autoimmune thyroiditis (4%), or were part of a consanguineous couple (4%). A significant percentage of the patients (28%) had experienced recurrent fetal/neonatal death or neonatal liver failure. The main prenatal ultrasound finding reported in the 74 cases was FGR (33% cases), followed by oligohydramnios (13% cases) and hydrops fetalis (13% cases). In 13% of the cases, the course of the pregnancy was described as uneventful. Other rare prenatal findings were fetal anemia, ascites, or abnormal fetal liver and spleen (Figure 2). MRI had been used as a prenatal diagnostic tool in only two cases (1.48%), at 32 WGA in both cases, and depicted an atrophic liver. In one case, the parents opted for ensanguine transfusion and intravenous immunoglobulin, with good results. In the other case, they opted for therapeutic interruption of pregnancy (TOP) [17]. MRI was performed in 11 cases (8.14%) to confirm extrahepatic siderosis in neonates, which was described mainly in the pancreas in nine cases (6.66%).

Most fetuses had an inauspicious prognosis, with the pregnancies ending in fetal death, perinatal death, or therapeutic interruption of pregnancy. Favorable evolution with treatment was reported for only 7% of the fetuses. Autopsy was the primary method used to confirm diagnosis. The postnatal treatment varied from chelation/antioxidant therapy, with reserve results, to ensanguine transfusion and intravenous immunoglobulin, with a favorable prognosis. When reported, the subsequent pregnancy had favorable evolution in cases of prenatal intravenous immunoglobulin (IVIG) treatment.

**Figure 3:**
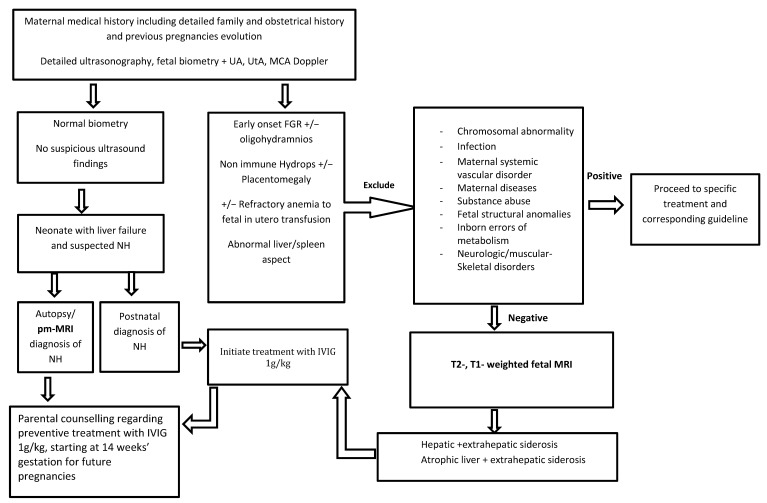
Prenatal diagnosis algorithm proposal for suspected neonatal hemochromatosis in the first affected pregnancy.

Following these results, in Figure 3, we propose a prenatal diagnosis algorithm for suspected NH in the first affected pregnancy.

## 4. Discussion

The objective of this paper was to systematically review the current literature regarding the main prenatal features of patients diagnosed with NH-GALD in order to create an algorithm for potential prenatal suspicion of this rare pathology characterized by a severe prognosis, in which selected fetuses will be referred for fetal MRI and the initiation of specific treatment where appropriate. While neonatal diagnosis has become more reliable, prenatal suspicion of NH in first affected pregnancies is exceptional. We reviewed 18 studies involving 74 fetuses diagnosed with NH and with confirmed extrahepatic siderosis, following which we could draw some conclusions regarding the prenatal features of fetuses with NH.

### 4.1. Etiology and Pathogenesis

Although the nature of the triggering antigen is still unknown, the central hypothesis regarding the pathogenesis of NH-GALD is focused on liver disease.

Fetal hepatocytes suffer injuries through the attack of membrane complexes resulting from transplacental transfer of maternal IgG antibodies [7], followed by impairment of iron homeostasis and siderosis in extrahepatic tissues [3]. Hemochromatosis often begins with iron buildup in hepatocytes, with the exception of Kupffer cells. The liver tissue of newborns with GALD is severely damaged, with hepatocyte loss leading to cirrhosis [1]. 

Iron deposition in diverse places is the ultimate phase in a multi-step process in which liver damage mediated by the complement may be the initial step [28].

Usually, extrahepatic siderosis is encountered in the pancreas, myocardium, thyroid, and salivary glands. Whittington et al. suggested that the possibility of alloimmune gestational should be considered since genetic causes for transmission do not align with known patterns of inheritance [7]. Even if NH is both congenital and familial, it is not hereditable [1].

During gestation, fetal iron levels are controlled by placental function, with trophoblastic tissue functioning as duodenal mucosa in tuning iron absorption and balancing the substantial maternal iron pool, thereby carefully regulating the fetal iron pool [2].

Fetal iron stores are regulated via the fetal hepcidin pathway [1]. The damage to fetal hepatocytes caused by maternal antibodies results in a decreased level of hepcidin, producing iron overload in the fetal liver [2]. However, recent data indicate that additional pathways, such as BMP/SMAD and JAK/STAT, can influence hepcidin expression [29].

Two main patterns of iron accumulation have been described: parenchymal iron overload and Kupffer cell hemosiderosis [30]. Parenchymal iron accumulation refers to accumulation in hepatocytes and bile duct epithelium, and in severe cases, portal fibrosis and eventual cirrhosis can be encountered [30].

Kupffer cell hemosiderosis refers to a pattern accumulation of hemosiderin in Kupffer cells and, in some cases, in the portal macrophages and endothelial cells, and secondary iron overload can be encountered. Moreover, mixed patterns of iron accumulation create even more issues, resulting in further difficulties in assessing these cases [30].

More recent data suggest that hepatic C5b-9 deposition could represent an important marker for GALD in an appropriate clinical situation, but the literature data are still contradictory [31].

An excellent study was conducted by Bonilla et al. in which liver tissues and extrahepatic tissues from GALD infants were investigated and compared with normal age-matched tissues. GALD-related liver injury may reduce hepcidin synthesis such that a cascade of reactions may follow, such as impaired placental iron flux feedback control. The authors pointed out that hepatic HAMP (hepatic antimicrobial protein) expression was reduced, and the pattern of extrahepatic siderosis is apparently determined by the capacity of various tissues to import non-transferrin-bound iron and not export cellular iron. Their findings suggest that NH is not primarily an iron overload disease [32].

An essential step is distinguishing the NH-GALD–related phenotype from other non-GALD disorders with different clinical presentations and prognoses. In order to accurately diagnose GALD, a comprehensive evaluation must be performed. Shanmugam et al. summarized the investigations that must be conducted to rule out other potential causes of a comparable phenotype [33]. For the other illnesses, it is postulated that fetal liver injury leading to poor regulation of placental iron flux causes NH. According to some authors, these causes account for approximately 2% of all NH cases [1].

The most frequent causes that should be taken into consideration for the differential diagnosis of NH in neonatal liver failure, other than gestational alloimmune, are presented in Table 2 [34,35,36,37].

Some writers emphasize the importance of the histologic findings and the function of autopsy, arguing that biological and molecular tests may be required to rule out alternative diagnoses, such as mitochondrial illnesses [28]. One particular finding in the histologic report is a grade of renal tubular dysgenesis with paucity or an absence of proximal tubules, explained as a developmental abnormality due to impaired hepatic angiotensinogen secretion [1,38], placing the onset of liver injury prior to the 24th week of gestation [2].

Even in suspected cases determined in utero, the detection of liver siderosis is insufficient for diagnosis due to the physiological presence of stainable iron in the neonatal liver, necessitating extrahepatic siderosis for confirmation or the detection of other specific markers that point to gestational alloimmune liver disease [7].

There have been reported cases of children with phosphatidylinositol N-acetylglucosaminyltransferase subunit A (PIGA) germ-line mutations presenting NH features, but their clinical course differs significantly. Therefore, PIGA gene testing is another option to be considered when evaluating newborns who present with NH [39].

### 4.2. Ultrasonography—The Key to Early Prenatal Diagnosis of Neonatal Hemochromatosis

FGR is one of the first manifestations visible in ultrasound evaluations that can be suggestive of NH, starting as early as the 18th week [17]. Since FGR has various causes, from utero-placental to genetic and infection, finding the right approach to correctly diagnose FGR-NH is a challenge.

Frequently encountered oligohydramnios is explained by renal tubular dysgenesis due to hepatocyte injury and secondary reduced angiotensinogen production as early as 20 to 25 weeks of gestation [1,38].

However, the FGR–oligohydramnios sequence related to NH often functions in diagnosis of exclusion during the first affected pregnancy but is also the most likely to be omitted since this clinical picture can be explained on the basis of more common causes. The process exclusion may be sufficiently long to result in an accurate verdict. Therefore, birth or fetal demise can occur, leaving the final diagnosis for neonatal or autopsy evaluation. Most of the time, these children are not born in tertiary centers. Until the transfer is successful and the treatment with immunoglobulin is initiated, the child’s prognosis may be compromised.

MRI is the only investigation technique that can guide the prenatal diagnosis algorithm to NH in a first affected pregnancy presenting FGR and oligohydramnios, since liver biopsy is not a viable option. Although expensive at first and not yet a part of the diagnosis algorithm of FGR, prenatal MRI can be used to elucidate a diagnosis of NH and detect placental dysfunction where Doppler flows are still normal [40], justifying the initiation of specific treatment.

Non-immune hydrops fetalis (NIHF) occurs in the end-stage state of 14 various diseases, according to a meta-analysis conducted by Bellini and colleagues in 2015 [41], with no identifiable primary cause in approximately 20% of cases [42]. The diagnostic algorithm used to determine the underlying cause of NIHF is a time trial challenge to assess whether there is a therapeutic solution to reverse the consequences. NIHF is a frequent prenatal finding in NH, which can most likely be explained by secondary hypoproteinemia [43]. After excluding immune hydrops fetalis, a hydropic and anemic fetus with associated hepatomegaly may raise suspicion of NH and justify a fetal MRI for diagnosis of exclusion.

As the fetus grows, unexplained hepatomegaly, sometimes accompanied by ascites, could be encountered during prenatal US surveillance.

In 1996, Achiron et al. examined a group of fetuses with various grades of hepatic hyperechogenicity. The authors identified several causes responsible for this particular imaging aspect, ranging from abnormal karyotype through to fetal infection to in utero vascular accidents [44]. They concluded that hepatic hyperechogenicity alone, with a lack of other markers, poses little clinical significance. Further investigation, such as by MRI, is required to diagnose NH accompanied by extrahepatic siderosis.

An aspect worth mentioning in terms of the difficulties of prenatal diagnosis of NH was described regarding a 33-week fetus presenting on US evaluation with hydrops, oligohydramnios, and placentomegaly. The re-evaluation of fetal ultrasound images resulted in identification of anemia and shrunken liver with multiple regenerative nodules, confirmed by MRI and autopsy [45]. Sometimes, re-evaluating the initial images and assessment may provide vital information for patient care.

**Table 2:** Differential diagnosis of neonatal hemochromatosis in neonatal liver failure.

Causes of Neonatal Hemochromatosis Phenotype Other Than Gestational Alloimmune Liver Disease	Prenatal Ultrasound Features	Investigations	Available Treatments	Perinatal Findings	Prognosis of Affected/Subsequent Pregnancies
1. Intrauterine infections Viral infections (e.g., viruses of the herpes family, cytomegalovirus, parvovirus, enterovirus, adenovirus) [33,46,47]	Fetal hydrops, cerebral ventriculomegaly, ± microcephaly, hyperechogenic fetal bowel, hepatosplenomegaly, cerebral periventricular echogenicity/intracranial calcifications, fetal cardiac arrhythmias, anemia, FGR, abnormal amniotic fluid volume, placental enlargement	Maternal serology AF PCR	Ganciclovir, valacyclovir, foscarnet, CMV hyperimmune globulin (not approved for routine use) Fetal intrauterine transfusion in case of an anemic and hydropic fetus	Fever, lethargy, abdominal distension, seizures	Poor/good
2. Metabolic disorders * Transaldolase deficiency [33,48,49]	FGR, hydrops fetalis, oligohydramnios, fetal distress, hepatosplenomegaly, hyperechogenic bowel mostly uneventful	DNA analysis (variants in the TALDO1 gene)	No established treatment in humans Benefit to supplementation with the glutathione precursor *N*-acetylcysteine in knockout mouse model	Jaundice, hypoglycemia	Poor/AR
Tyrosinemia type I [33,50,51]	Mostly uneventful	Blood succinylacetone level (screening marker) Molecular testing of *FAH*, encoding FAH	2-[2-Nitro-4-trifluoromethylbenzoyl]-1,3cyclohexanedione	Jaundice, hypoglycemia	Good with early treatment/AR
Galactosemia/hereditary fructose intolerance [33,52]	Mostly uneventful	Measurement of GALT enzyme activity in red blood cells (absent or significantly decreased GALT gene analysis)	Galactose-restricted diet	Jaundice, hypoglycemia	Good with early diagnosis and diet/AR
Niemann Pick type C [53,54,55]	FGR, oligohydramnios, hepatomegaly, splenomegaly, hydrops fetalis, hygroma	Genetic testing (NPC2 gene)	No curative treatment Iminosugar N-butyldeoxynojirimycin Management of the neurological disease	NLF without a free interval after birth	Poor/AR
Urea cycle disorders [56]	Mostly uneventful, premature birth	Mutation analysis using DNA from CVS or AFC Citrulline and ASA determinations in AF for prenatal diagnosis of ASSD and ASLD	Nitrogen scavengers benzoate and phenylacetate arginine and/or citrulline N-Carbamylglutamate	Neurological impairment, seizures, hyperammonemia, lethargy, anorexia, hyper-/hypoventilation	Outcomes are generally poor without early treatment/good with strict supervision
Disorders of glycosylation [57]	FGR, skeletal anomalies, pericardial effusion	Multiple, DNA analysis	No curative treatment Organ transplantation to relieve selected symptoms Promising new monosaccharides (particularly mannose and galactose) and chaperone therapies	Axial hypotonia, hyperreflexia, esotropia, development delay. A non-fatal neurological form and a multivisceral neurological form.	Poor/most forms of CDG are inherited in AR
Mitochondrial cytopathies [58]	Usually uneventful	Gene sequencing Mutation in the mitochondrial DNA or nuclear DNA	No curative treatment	Hypotonia, seizures, abnormal liver function, impaired feeding	Depends on how many organ systems and tissues are affected/AR, AD Mitochondrial Random mutations
4. Primary bile acid disorders [59]	Usually uneventful	HSD3B7 gene for 3β-HSD deficiency; AKR1D1 gene (formerly SRD5B1) for Δ4-3-oxoR deficiency.	Cholic and chenodeoxycholic acid	Neonatal hepatitis, cholestasis, diarrhea, steatorrhea, malabsorption, progressive neurological disease, cirrhosis	50% mortality without treatment/AR
5. Genetic—syndromic Trisomy 21, trisomy 18, cerebrohepatorenal syndrome, renal tubular dysgenesis syndrome, Donohue syndrome, trichohepatoenteric syndrome [3]	FGR, diverse structural anomalies	Karyotype/gene testing	No curative treatment	Clinical presentation may vary widely from single anomaly to multiple malformations	Poor/depends on parental genetic status
6. Rhesus incompatibility, AB0-incompatibility, congenital hemolysis [60]	Hydramnios, FGR, hydrops fetalis, hepato-splenomegaly, ascites	Clinical and laboratory assessment (Coombs test, qPCR to detect RHD gene in cfDNA)	The main principle is the prevention of maternal sensitization anti-D immune globulin	Anemia, hyperbilirubinemia, thrombocytopenia, neutropenia, kernicterus, hypomagnesemia, neurological complications	Generally good/good with early administration of Rh IgG if the fetus is Rh positive
7. Hematological disorders Hemophagocytic lymphohistiocytosis [61]	No data found regarding prenatal ultrasound findings of HLH	Hematological and imagistic tests Evaluations for infections and malignancies Rapid screening tests for genetic HLH	Immunosuppressive and chemotherapeutic treatment	Fever, splenomegaly, cytopenias, hypofibrinogenemia, ±hypertriglyceridemia, hyperferritinemia, hemophagocytosis, low NK activity, high concentration of soluble interleukin 2receptor (sCD25/sIL-2R)	High mortality rate/various genetic causes of predisposition
8. Exposure to toxic agents Pyrrolizidine [62,63]	No data found regarding prenatal ultrasound findings of pyrrolizidine exposure	Classic methods (urine colorimetric screening test), chromatography, mass spectrometry, immunoassays	Stopping the exposure, symptomatic treatment, anticoagulant therapy, transjugular intrahepatic portosystemic shunt, liver transplant, defibrotide	Abdominal pain, jaundice, hepatomegaly, ascites, weight gain	Chronic exposure causes hepatic sinusoidal obstruction syndrome/good
11. Exogenous iron overload [64]	No data found regarding prenatal ultrasound findings of exogenous iron overload	Mutations in the genes for transferrin receptor 2 (*TfR2*) or ferroportin (*SLC40A1*) Fasting serum testing for ferritin, total iron-binding capacity, transferrin saturation, iron levels and metabolic panel, including hepatic enzymes MRI	Phlebotomy Iron chelators: deferoxamine and deferasirox Orthotopic liver transplantation Diet modification	Fetal growth retardation, aminoaciduria, cholestasis, liver hemosiderosis, hyperferritinemia, hypotransferrinemia, increased transferrin iron saturation, and free plasma iron, lactic acidosis	Good if no end-organ disfunction/AD

* Some of the metabolic disorders are also genetic metabolic disorders. AF = amniotic fluid; CDG = congenital disorders of glycosylation; CVS = chorionic villous sample; AFC = amniotic fluid cells; ASA = argininosuccinate; ASSD = argininosuccinate synthetase deficiency, citrullinemia type I; ASLD = argininosuccinate lyase deficiency; UCD = urea cycle disorders; HLH = hemophagocytic lymphohistiocytosis; AR = autosomal recessive disorder; AD = autosomal dominant; FGR = fetal growth restriction; MRI = magnetic resonance imaging; NLF = neonatal liver failure; NK = natural killer.

This particular aspect of the liver, similar to that in adult cirrhosis, is challenging to evaluate in fetuses. Therefore, additional investigations such as fetal MRI are needed to confirm any suspicions.

Unexplained fetal anemia, refractory to fetal in utero transfusion, is another US finding that can be prenatally detected without difficulty after introducing middle cerebral artery systolic peak measurement in routine practice. This could raise suspicion of NH [9].

Encountering NH during the first pregnancy in consanguine couples is an aspect for which clinical significance is yet to be revealed [12]. Another observation that does not fit the NH prenatal pattern is the different liver damage grades observed in some twin pregnancies [1]. In unexplained cases of fetal death, NH should be considered as one of the differential diagnoses, although cautiously, considering that US signs of NH are insufficient in the first affected pregnancy [3].

### 4.3. Magnetic Resonance Imaging—New Horizons for Prenatal Diagnosis and Follow-Up of Neonatal Hemochromatosis

Although there is evidence of its effectiveness [65], MRI is scarcely used in prenatal NH diagnosis. It is mainly utilized as a research tool rather than a diagnostic tool in neonates [45]. Quantitative assessment of organ iron deposition for the adult population is available using T1 or T2* relaxometry, and this can be applied in pediatrics. However, there is still no available normative data on iron quantity in various neonate tissues [4,66].

Fetal MRI in GALD could help assess clinical responses to in utero treatment [67]. The methods used encompass a qualitative assessment of the signal intensity of the respective organs on T2, T2*, T1, and GRE (gradient recalled echo) sequences.

However, depicting the liver overcharged with iron is insufficient for diagnosis, even if other immune manifestations, such as hydrops fetalis, are present. Extrahepatic siderosis is considered a fundamental criterion for diagnosis, but a GALD hypothesis should be raised, even in its absence, when considering confirmed affliction in previous pregnancies. Recent studies emphasize this, recommending that prenatal treatment should not be postponed when there is no finding of extrahepatic non-reticuloendothelial siderosis but there are other specific markers pointing to gestational alloimmune liver disease [31].

Even if fetal organ iron deposition can be demonstrated by MRI, it may not always be present at the moment of examination, as hypothesized as far back as 1990 by Hoogstraten [67]. Cases have been reported with an atrophic liver both with and without observable extrahepatic iron impregnation. On the other hand, liver siderosis could be absent, as the targeted hepatocytes could have already been destroyed [17]. In these cases, the presence of extrahepatic iron impregnation could help with diagnosis [7].

There have been reports of several MRI sequences that can show organ iron overload in the fetus. In the first case of intrauterine hemochromatosis diagnosed with MRI in 1993, Marti-Bonmati et al. used a T2* sequence. Extremely low hepatic parenchymal signal intensity (SI) was shown compared with that of fetal and maternal fat tissue [68].

Presently, fast, single-shot T2-weighted sequences are the primary tool in fetal MRI and the first to raise suspicion of liver iron deposition. The affected fetal liver shows an SI drop that is even more conspicuous when compared with the fetal spleen or maternal liver SI.

The same pattern of signal intensity alteration seen in neonates with NH has been described in fetuses with liver siderosis in a few case presentations—lower median SI of the liver on in-phase sequences compared with the opposed-phase SI.

The in-phase/opposed-phase SI ratio (SIR) in fetal NH has been reported as approximately 0.5 in an untreated fetus and 0.78 in a fetus with prior IVIG treatment. In one case of prior clinically and histologically proven GALD, Sasaki et al. also calculated the SIR of the fetal liver, spleen, and pancreas to the fetal iliopsoas muscle. They obtained values of 0.7, 1.36, and 1.74, respectively, suggesting that iron deposition in this treated case was limited to the liver [66,69].

Organ iron load quantification could be achieved with MRI T2* relaxometry techniques, as described in 2013 by Schoennagel et al. in a fetal sheep model and a human neonate with hemochromatosis [70]. Sethi et al. achieved further progress in a future quantitative assessment of fetal organ iron load by setting the baseline for quantifying the T1 and T2* relaxation times of fetal tissues in uncomplicated pregnancies at the gestational age of 28–38 weeks [71].

A French retrospective multicentric study of the importance of autopsy and auto-immune maternal manifestations in neonatal hemochromatosis observed that, in fetuses, iron storage was more frequent in the thyroid than in the pancreas [28]. As far as we know, there has been no reported MRI finding (or search) of fetal thyroid iron load in hemochromatosis. The thyroid signal intensity in 3D GRE T1 was evaluated in seven fetuses with hypothyroidism and 17 fetuses with normal thyroid function (GA of 28–41 weeks). The mean and maximum SIRs in fetuses with a normal thyroid was 1.85 ± 0.20 and 2.61 ± 0.39 [72]. This finding could be a reference for normal fetal thyroid SI on GRE T1 sequences when assessing fetuses with hemochromatosis.

Extrahepatic siderosis can be detected non-invasively by a multi-echo gradient recalled echo T2*-weighted MRI sequence within hours of birth and graded as mild, moderate, or severe based on the echo in which organs start showing darkening/a signal drop [4,66].

Another essential step in confirming NH where MRI may play an important role is postmortem evaluation. In selected cases where parents’ religious or moral views prevent a classical autopsy evaluation, a virtual evaluation by postmortem MRI might be a valuable alternative with a high grade of acceptability from the genitors. Postmortem MRI can easily depict liver siderosis [73] or extrahepatic siderosis and can therefore be the necessary justification for further investigations through a guided liver biopsy ±C5b-9 immunostaining.

### 4.4. In Utero Treatment and the Prevention of Neonatal Hemochromatosis Sequelae

In 2004, Whittington proposed the antenatal administration of a high dose of intravenous immunoglobulin (IVIG), starting in the second trimester, for women with a previous pregnancy with NH [7]. Today, treatment with IVIG has practically changed the prognosis of affected women, allowing them to bear a healthy child after a pregnancy with NH [74,75]. The current guidelines recommend administering IVIG to secondary pregnancies at a dose of 1 g/kg at 14 and 16 weeks and then weekly from 18 weeks of gestation. The treatment significantly improved disease evolution with absent US manifestations and a better life expectancy than in untreated pregnancies [67]. However, IVIG treatment is expensive; availability can be limited in some countries, and severe adverse effects may present [75].

The mechanism by which IVIG administration improves fetal evolution remains unclear, but some theories have been proposed. One considers the dilution of maternal antibodies directed against the fetal liver antigens as a possible mechanism [3]. Another proposes that IVIG may block placenta receptors and reduce the placental transmission of maternal antibodies to the fetus, or that IVIG may even block Fc receptors on the macrophages in the fetal circulation, limiting the destruction of fetal hepatocytes sensitized by maternal antibodies [3].

### 4.5. Future Diagnostic Perspective

An in utero diagnosis of NH would be a remarkable achievement, as early diagnosis would prove useful for fetal treatment therapy. Based on the wide range of risks posed by a fetal liver biopsy and its relatively poor specificity, a more effective method is required [75].

**Figure 4:**
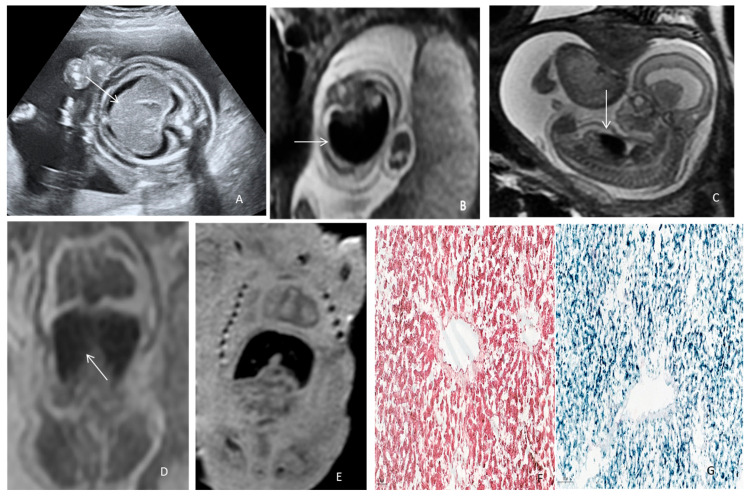
Neonatal hemochromatosis in a hypotrophic fetus with idiopathic non-immune hydrops. (**A**) Ultrasound axial abdominal section depicting hepatosplenomegaly (arrow), ascites, and subcutaneous edema at 17 WGA. (**B**) Prenatal MRI 3T, T2 WI depicting fetoplacental anasarca and the liver with axial T2 hypersignal and (**C**) sagittal section (arrow) at 18 WGA. (**D**,**E**) Virtual autopsy using postmortem MRI, T2WI, and T1WI in the coronal plane depicting fetal liver with low signal (arrow), indicating the presence of iron at 21 WGA. (**F**) Hematoxylin–eosin staining (7×) showing two centrilobular veins and cords of hepatocytes. (**G**) Perls staining showing hemosiderin deposits (blue) in liver parenchyma (8×).

We consider that a prenatal MRI should be considered earlier in cases presenting FGR with normal Doppler parameters or unexplained non-immune hydrops fetalis, refractory anemia to fetal in utero transfusion, or liver abnormalities in the diagnosis algorithm. Furthermore, for parents that refuse to allow a classical autopsy of the fetus/neonate, postmortem MRI is a non-invasive solution that can easily identify liver siderosis and guide biopsy. To illustrate this idea, a figure of a fetus with confirmed NH investigated using both in vivo and postmortem MRI is rendered in Figure 4.

### 4.6. Limitations

NH-GALD is a rare, severe disorder that results in fetal loss or neonatal death due to liver failure; therefore, the present study is limited by the small number of included studies and the retrospective nature of the reported cases.

## 5. Conclusions

Despite the strong impact of this condition on present and future pregnancies, the prenatal findings that might suggest fetal NH are often nonspecific. MRI should be indicated in selected cases of unexplained FGR, oligohydramnios, fetal hydrops, fetal hepatomegaly, and ascites or anemia, usually after all the other more frequently encountered conditions have been ruled out. MRI can detect iron overload in the liver and extrahepatic siderosis, confirming NH. For the parents that refuse a fetal autopsy, postmortem MRI should be available for all cases of unexplained fetal and newborn deaths with liver failure, as it can easily detect liver siderosis and guide future investigations. Once the diagnosis is made, the administration of specific IVIG should be initiated immediately after diagnosis, allowing for a much better prognosis. C5b-9 staining of the tissues can confirm the diagnosis from the liver biopsy or autopsy specimen.

In subsequent pregnancies, women with a previously NH-affected fetus should be carefully evaluated, and high-dose IVIG treatment should be started at the end of the first trimester, as evidence shows significant improvement in the disease evolution compared with untreated pregnancies. In neonates, exchange transfusion and a high dose of IVIG is the preferred treatment to remove the maternal NH-associated antibody and block its action, including complement activation.

## Figures and Tables

**Table 1:** Literature review of the cases diagnosed with neonatal hemochromatosis, with confirmed extrahepatic siderosis (1993 to 2021) and maternal medical and obstetric history, prenatal findings, postpartum outcome, and subsequent pregnancy outcome.

Article, Year	Number of Cases Included	Gravidity (G) (Range), Weeks of Gestational Age (WGA) at Delivery, Mean		Women with Recurrent fetal Death or NLF	Consanguinity/ Autoimmunity	Prenatal Finding	Weight (g)	Diagnosis Confirmation	Evolution of the Case	Treatment	Evolution of Subsequent Pregnancy
1. Mugarab-Samedi et al., 2021 [11]	1	4G, 29 WGA		yes	no	FGR, anhydramnios	840 g	autopsy	death	Glucose, antibiotics, fresh frozen plasma, cryoprecipitate, platelets, and vitamin K, laparotomy for suspected necrotic bowel	Favorable with IVIG
2. Mude et al., 2020 [12]	1	1G, 38 WGA		no	consanguineous couple	FGR	2600 g	buccal and liver biopsy	death	Fresh frozen plasma, chelation-antioxidant therapy ET + IVIG	NM
3. Maisonneuve et al., 2019 [13]	1	1G, 34 WGA		no	no	FGR, anhydramnios, hydrops, small liver, anemia, white matter anomalies	NM	autopsy	TOP	Intrauterine transfusion for moderate anemia at 8.8 g/dL at 32 WGA	NM
4. Zaharie et al., 2018 [14]	1	2G, 37 WGA		no	no	FGR, OA, normal middle cerebral to umbilical artery Doppler ratio, no notching on the uterine artery, but a constant slightly tachycardic fetal heart rate	2300 g	autopsy	death	Iron chelation-antioxidant, IVIG	NM
5. Darouich et al., 2018 [15]	1	3G, 37 WGA		yes	gestational diabetes	OA, hydrops, ascites, enlarged liver and inhomogeneous echogenicity of the hepatic structure with reticulonodular aspect, cholelithiasis	5000 g	autopsy	stillbirth	–	NM
6. Babor et al., 2018 [16]	1	6G, 38 + 6 WGA		yes	no	FGR	2200 g	oral mucosa biopsy	favorable	Fresh frozen plasma, ET + IVIG	NM
7. Sciad et al., 2018 [17]	4	NMG, 21WGA, 30WGA 32WGA 34WGA		yes in 2 cases	no	4 FGR, 4OA, 3Hydrops, 1 hepatosplenomegaly, 3oedematous placenta	NM	2MRI (f) 3 at autopsy 1 liver biopsy after birth	1 favorable with IVIG 1 stillborn 1 postpartum death, 1 TOP at 32 WGA	Prenatal IVIG in the survivor case	NM
8. Da Rocha et al., 2017 [18]	1	1G, 31 + 4 WGA		no	no	FGR, OA	850 g	MRI (p) autopsy	death	NM	NM
9. Lopes et al., 2015 [19]	1	1G, 32 WGA		no	no	FGR, OA, hyperechogenic liver lesions in the peripheral parenchyma, edematous placenta	1520 g	autopsy	death	frozen plasma, peritoneal dialysis	NM
10. Rodrigues et al., 2015 [20]	12	MNG, Mean 37 WGA (range 32–40)		yes in 5 cases	1 SLE	8 FGR, 2OA, 8 normal, 2 edematous placentas	2529 g (range 1640–3700 g)	2 MRI (p) biopsy and autopsy	10 deaths 2 survived with chelator	5 no treatment 7 treated with chelation/antioxidant therapy	NM
11. Heissat et al., 2014 [21]	7 fetuses 22 neonates	5 G1 17 G2–G13 Fetuses: Mean ± SEM 29.6 ± 0.9 WGA Neonates: Mean ± SEM 32 ± 37WGA		Yes in 7 cases	1consanguinity 1 SLE, 2 AT 1 vasculitis	14 FGR 13 OA 10 hydrops 9 normal	NM	5 MRI (p) 4 liver biopsies, 24 autopsies	7 stillborn death	NA	Three women were treated with IVIG during subsequent pregnancies and resulted in live births with an uneventful neonatal course
12. Baruteau et al., 2014 [22]	8	NM G 35 WGA (range 29–41)		NM	1 AT 1 SLE	5 FGR, 7OA	NM	2 MRI (p) autopsies	3 stillborn (27,28,31 WGA) 6 neonatal untreated	IVIG	Favorable without recurrence in subsequent pregnancies
13. Amann et al. 2011 [9]	2	IG, 32 WGA and 23 WGA		yes	consanguineous couple	FGR, ascites, severe anemia, aortic coarctation, meconium peritonitis	1660 g	liver biopsy	death at 4 months of age	surgical treatment for meconial peritonitis Iron chelation with an antioxidant cocktail	TOP for same clinical spectrum including aortic coarctation at 23 WGA (confirmed NH)
14. Tanaka et al., 2011 [23]	2	4G, 36 WGA and 30 WGA		yes	no	FGR, OA in both cases	1000 g, 500 g	autopsy	death	NM	Favorable with prenatal IVIG treatment
15. Çakır et al., 2011 [24]	1	2G, 38 WGA		no	no	uneventful	3150 g	MRI (p) Liver biopsy at 6 months	favorable	ET + IVIG	NM
16. Kassem et al., 1999 [25]	2	5G, 32 WGA and 34 WGA		yes	no	FGR, OA, hydrops, anemia	1960 g, 2400 g	autopsy	death	ET	NM
17. Verloes et al., 1997 [26]	2	1G 35 WGA and 37 WGA		yes	no	FGR	1410 g, 1860 g	autopsy	death	NM	NM
18. Wisser et al., 1993 [27]	1	1G, 36 WGA		no	no	FGR, hydrops, ascites, hepatosplenomegaly	3200 g	autopsy	death	none	One healthy boy at term after 18 months without prenatal treatment
Total	74 cases	16% G1	84% ≥G2	Women with recurrent fetal death or NLF 28%	No maternal pathology 86% Consanguineous couple 4% SLE 4% AT 4% Vasculitis 2%	Uneventful 13% FGR 33% OA 25% Hydrops 13% Ascites 3% Abnormal liver/spleen aspect 4% Anemia 2% Other 2%	Mean 2028 ± SD 1090.67 g Range 500–5000 g	51% confirmed at autopsy	93% stillborn, perinatal death or TOP 7% favorable evolution with treatment	chelation/antioxidant therapy ET + IVIG none	When mentioned, the subsequent pregnancy had favorable evolution with prenatal IVIG treatment
		Mean 33 (SD ± 4.57) WGA Range 21–40 WGA									

NLF = neonatal liver failure; SLE = systemic erythematosus lupus; AT = autoimmune thyroiditis; FGR = fetal growth restriction; OA = oligoamnios; TOP = therapeutic interruption of pregnancy; ET = ensanguine transfusion; IVIG = intravenous immunoglobulin; NM = not mentioned; f = fetal; p = postnatal.

## Data Availability

The data presented in this study are available on request from A.S.
